# The fetus/infant - mother as a co-evolving dyadic system and the development of attachment styles: an active inference perspective

**DOI:** 10.3389/fpsyg.2026.1836911

**Published:** 2026-06-18

**Authors:** Erica Santaguida, Giuseppe Pagnoni, Massimo Bergamasco

**Affiliations:** 1 Institute of Mechanical Intelligence, Sant’Anna School of Advanced Studies, Pisa, Italy; 2 Department of Biomedical, Metabolic and Neural Sciences, Section of Physiology and Neuroscience, University of Modena and Reggio Emilia, Modena, Italy

**Keywords:** active inference, attachment, internal working models, predictive processing, prenatal development

## Abstract

The mother–fetus/infant dyad constitutes a uniquely asymmetric biological and psychological system whose co-evolutionary dynamics we propose are most coherently understood within the Active Inference Framework (AIF). We hypothesize that the development of attachment styles emerges from the progressive construction of a generative model in the fetus and infant, shaped by the precision and content of predictions learned through early dyadic interactions — beginning already during the gestational period. Drawing on the Free Energy Principle and its neuroscientific and mathematical foundations, we propose that internal working models (IWM), as originally conceived in attachment theory, are best reinterpreted as hierarchical generative models that develop through reciprocal inferential exchanges across the Markov blankets of two asymmetrically coupled agents: the mother and the fetus/infant. The quality and consistency of maternal caregiving determines the precision of the infant’s predictions, which in turn organizes the attachment system along the axes of security and behavioral organization. This framework integrates neurobiological, epigenetic, cognitive, and developmental perspectives under a single first-principles account, offers novel testable predictions, and opens new directions for clinical translation, particularly regarding early intervention and intergenerational transmission of attachment.

## 1 Introduction

### 1.1 Attachment theory: foundations and open questions

Bowlby’s (1969/1982) pioneering work introduced attachment theory, which explores the emotional bond between mother and child based on ethological studies and systems theory. Attachment is an innate behavior to maintain proximity to the primary caregiver for protection against external disturbances. This is achieved and maintained through attachment behaviors such as smiling, crying, sucking, clinging, and locomotion, which manifest from the early months of life and evolve in complexity from early infancy. The theory views attachment as a control system that uses feedback mechanism to regulate closeness to the caregiver, providing a sense of security (Bretherton, 1980). Crucially, Bowlby conceptualized this control system as cognitively mediated: it monitors the environment, generates internal representations of contingencies, and activates or inhibits attachment behaviors. The intensity and frequency of these behaviors depend on factors like the child’s tiredness, hunger, stress, potential danger, and the caregiver’s availability.

Central to Bowlby’s architecture is the concept of an internal working models (IWM): dynamic, hierarchically organized cognitive structures representing the attachment figure, the Self, and the relationship between them (Bowlby, 1969/1982). Inspired by Craik’s (1943) notion of operational models, IWM serve as predictive maps that enable the child to anticipate caregiver behavior, evaluate environmental contingencies, and regulate attachment-related affect and behavior accordingly. Although a dominant model is always operative, alternative models of the same figure may coexist, often at lower levels of conscious accessibility (Bowlby, 1969/1982).

Ainsworth et al.’s (1978) systematic observations revealed three attachment styles that are clearly observable around the second year of life, as relatively stable patterns of behavior, emotions, and thoughts that develop based on the quality of interpersonal relationships with the primary caregiver: secure attachment, characterized by a high level of synchronization (Schore, 2021) between the child’s and the primary attachment figure’s behavioral systems, allowing the child to develop trust in others and in herself; insecure anxious attachment, marked by hyperactivation of the attachment system relative to other classes of behavior, such as exploration, and associated with a sense of poor self-efficacy; and insecure avoidant attachment, characterized by the suppression of attachment behaviors even in times of need, often with a counterbalancing prevalence of exploratory behavior, and by a working model in which the representation of Self may remain relatively positive while others and the world are characterized by low trust. Despite various classifications of attachment styles (e.g., Main and Solomon, 1990; Crittenden and Landini, 2011), reference in the present theoretical work will be made to these three main styles.

Building on foundational distinctions, numerous studies have explored attachment theory, focusing on the internal processes that establish and maintain attachment bonds (for a review see Santaguida and Bergamasco, 2024). Genetic research has shown that genetic polymorphisms can influence the development of specific attachment styles, either reducing or enhancing the impact of relationship quality (Craig et al., 2021; Rasmussen and Storebø, 2021; Van Ijzendoorn and Bakermans-Kranenburg, 2006; Bakermans-Kranenburg and van IJzendoorn, 2011; Erkoreka et al., 2021). Additionally, early social environment characteristics can affect genetic configurations through epigenetic processes, suggesting that the environment significantly shapes attachment style beyond innate predispositions (Van Ijzendoorn et al., 2010; Murgatroyd et al., 2015; Bakermans-Kranenburg and van IJzendoorn, 2011). Studies developed from the neuroscientific perspective showed how the early experience, e.g., of neurophysiological synchronization (Schore, 2021), leads to functional and structural neural changes, with long-lasting effects (Long et al., 2020). Cognitive developmental sciences have detailed how mental representations are organized according to attachment experiences (Sherman et al., 2015).

Another important development in attachment theory concerns the research into the prenatal origin of attachment. Research suggests that the gestational period may already be relevant to the foundations of the future attachment relationship. The mother’s bond with the fetus can predict the future quality of their relationship (Cranley, 1981; Zimerman and Doan, 2003; Smorti et al., 2020). From the opposite perspective, an open question is whether the psychological functions of the fetus and its behavioral affordances are sufficient to lay the essential foundations for a bond. Studies on fetal and perinatal cognitive capacities (e.g., Kadic et al., 2009; Kadic and Kurjak, 2018; Long et al., 2020; Gorina-Careta et al., 2024) suggest that prenatal development already includes forms of learning, early sensory discrimination, and rudimentary expectation formation. At the same time, the fetus exists in a condition of continuous proximity, or co-embodiment, with the mother (Ciaunica et al., 2021), and therefore does not yet require behavioral systems for regulating physical proximity in the way the postnatal infant does. This makes it theoretically plausible that some antecedents of later internal working models may begin to emerge during gestation, potentially preparing the organism for the social conditions it is likely to encounter after birth. In this sense, the hypothesis remains compatible with Bowlby’s view that a working model, to be adaptive in novel situations, must be able to anticipate conditions that have not yet been directly experienced (Bowlby, 1969/1982). However, this possibility should still be regarded as speculative rather than empirically established, since current evidence does not demonstrate the presence of fully developed attachment-related internal working models in utero. Despite these advances, the theoretical landscape remains fragmented. Multiple scientific perspectives — genetic, epigenetic, neuroscientific, and cognitive — converge on the attachment system from distinct vantage points but lack a shared formal language capable of integrating them into a coherent, mechanistic account. A further integrative challenge concerns the prenatal period: emerging evidence suggests that the foundations of attachment may be laid before birth, during a phase of intimate biological co-embodiment between fetus and mother (Ciaunica et al., 2021; Quintero and De Jaegher, 2020). Yet the conceptual tools of classical attachment theory — developed primarily to describe postnatal behavior — are ill-suited to address this earliest developmental phase.

At the same time, the prenatal period remains theoretically contested. In part, this reflects an ongoing debate over whether “prenatal attachment” should be understood mainly as parental bonding toward the fetus, or whether the concept can be extended in a stronger Bowlbian sense to developmental processes continuous with postnatal attachment regulation (Brandon et al., 2009; Trombetta et al., 2021). A related debate concerns whether the available prenatal evidence is better interpreted in terms of sensory learning, co-homeostatic regulation, and embodied self-organization, rather than as evidence for attachment-related internal working models in any strong sense (Ciaunica et al., 2021; Quintero and De Jaegher, 2020). The present proposal does not seek to resolve these debates definitively; rather, it advances the hypothesis that Active Inference provides a useful integrative framework for interpreting how prenatal and early postnatal processes may become developmentally continuous.

### 1.2 The need for a unifying theoretical framework

Recent reformulations of IWM theory have moved toward embodied cognition perspectives (Petters and Waters, 2014; Beckes et al., 2015), emphasizing that attachment dynamics such as proximity, warmth, and separation are grounded in bodily and sensory experience rather than abstract symbolic computation. Atzil et al. (2018) have further proposed that dyadic interactions serve allostatic regulatory functions, with the caregiver acting as an external regulator of the infant’s homeostatic equilibrium. These reformulations bring attachment theory into closer contact with contemporary neuroscience and with frameworks that take embodiment seriously, but they do not yet provide a unified formal account.

Several theoretical frameworks have sought to address aspects of early attachment development that classical attachment theory leaves underspecified. The Polyvagal Theory (Porges, 1995, 2011) offers a neurophysiological account of the autonomic substrates of social engagement, highlighting the role of vagal regulation in proximity-seeking and caregiving behaviors — a dimension that complements attachment theory but does not provide a formal account of representational development or dyadic co-evolution. Predictive coding frameworks (Clark, 2013; Hohwy, 2013) offer a computational perspective on perception and belief updating that maps naturally onto the concept of IWM, yet they are primarily perceptual theories and do not extend readily to the action-oriented, policy-selecting dynamics that characterize the attachment behavioral system. Enactive approaches (De Jaegher and Di Paolo, 2007; Quintero and De Jaegher, 2020) emphasize participatory sense-making and the relational constitution of agency, capturing important phenomenological dimensions of the mother–fetus/infant dyad, but they resist formalization in ways that limit their capacity to generate precise, testable predictions. Allostatically-oriented reformulations of attachment (Atzil et al., 2018; Feldman, 2017) bring the framework into closer contact with interoceptive and homeostatic regulation, but lack a unifying computational principle. We acknowledge that each of these frameworks captures genuine and important aspects of early relational development, and that the AIF account proposed here is not without its own limitations (Colombo and Series, 2012; Colombo, 2022). Nonetheless, we argue that AIF is uniquely suited to serve as an integrative framework because it subsumes perception, action, and homeostatic regulation under a single formal principle — free energy minimization — while providing a mathematically tractable account of how generative models develop, update, and organize behavior across multiple timescales.

We propose that the Active Inference Framework (AIF), rooted in the Free Energy Principle (Friston et al., 2006; Friston, 2010; Santaguida and Bergamasco, 2022), provides such an account. The AIF offers a mathematically precise, biologically grounded, and computationally tractable framework for understanding how living systems — from single cells to social groups — maintain their organization by minimizing prediction error (or, formally, variational free energy). Its core constructs — generative models, Markov blankets, precision-weighted prediction errors, and the distinction between pragmatic and epistemic action — map naturally onto the key concepts of attachment theory, while also capturing aspects of prenatal development, epigenetic regulation, and neurobiological organization that fall outside the scope of classical attachment accounts.

The present paper develops this proposal in four steps. We first introduce the core concepts of the AIF necessary for the theoretical argument. We then reconceptualize the mother–fetus/infant dyad as a system of two asymmetrically coupled generative models. Next, we analyze the convergence between IWM and the generative model construct, arguing that AIF provides a principled reinterpretation of IWM development. We then propose that the precision of dyadic predictions, as learned through repeated early interactions, is the proximal causal factor driving attachment style differentiation. Finally, we consider the complementary relationship between attachment and exploratory behavior through the lens of pragmatic and epistemic policies, and discuss how attachment style influences personality development. Throughout, we are careful to distinguish between well-supported empirical claims and theoretical hypotheses that remain to be tested.

## 2 Core concepts of active inference

Active Inference is a theoretical framework that has generated far-reaching reformulations of action, perception, and decision-making by grounding them in a single organizing principle: the minimization of variational free energy (Friston et al., 2006; Friston, 2010). Its broad explanatory scope, spanning the relationship between organism and environment from subcellular to social scales, makes it particularly suited to the integrative purposes of the present work.

### 2.1 Free energy minimization and the generative model

All living organisms face a fundamental imperative: to maintain their biological and psychological organization against the thermodynamic tendency toward disorder. In the AIF, this imperative is formalized as the minimization of *surprisal* — the long-run average discrepancy between an organism’s expected and actual sensory states — which, if unchecked, would dissolve the organism’s characteristic organization (its phenotype). Because surprisal is generally intractable, living systems minimize a computable upper bound on it known as *variational free energy* (Friston, 2010). This provides a mathematically precise account of how organisms self-organize and adapt.

The mechanism through which free energy is minimized involves a *generative model* — the full set of probabilistic beliefs that an agent embodies about how its environment produces the sensory data it receives. This model encodes both a representation of the world (what causes what) and a representation of the agent itself (how its actions alter sensory inputs). Generative models in AIF are hierarchical: lower levels encode rapidly fluctuating sensory signals, while higher levels encode slower, more abstract regularities (Parr et al., 2022). Perception, in this framework, is the inferential process of updating the model to best explain incoming sensory data — a view with deep roots in von Helmholtz’s notion of perception as unconscious inference. Action, complementarily, is the process of changing the world to match the model’s predictions. Both perceptual inference and active inference serve the shared goal of minimizing the discrepancy between predictions and sensory evidence (Friston, 2010).

### 2.2 Precision, prediction error, and belief updating

A critical feature of the AIF that is central to the present theoretical proposal is the concept of *precision*: the confidence (inverse variance) assigned to predictions and to sensory signals, respectively. Precision is not fixed but is itself estimated and updated in a hierarchical fashion (Parr et al., 2022). When precision of the predictions is high, the agent trusts its predictions strongly and requires large prediction errors to update its model. When precision is low, the agent is uncertain and remains highly sensitive to incoming sensory evidence. Crucially, the relative precision of top-down predictions versus bottom-up sensory signals determines whether free energy is minimized by updating the model (perception) or by acting to change the world (action). This precision-weighting mechanism provides a natural account of attention, as the selective amplification of certain prediction errors over others (Hohwy, 2013).

For agents capable of planning and decision-making, action selection involves minimization of *expected* free energy — the free energy anticipated under a given policy (sequence of actions) in the future. Expected free energy integrates both pragmatic value (the degree to which a policy brings about preferred, biologically and psychologically valued states) and epistemic value (the degree to which a policy reduces uncertainty about the model of the world by gathering informative sensory data) (Parr et al., 2022). This formalization captures the tension between exploitation and exploration that is central to adaptive behavior.

### 2.3 The Markov blanket and the boundary of the self

In defining the boundary between an agent and its environment, the AIF relies on the concept of the *Markov blanket* — a statistical structure that formally separates internal states of the agent from external environmental states, while connecting them through sensory states (inputs from the environment) and active states (outputs to the environment) (Kirchhoff et al., 2018). The internal states of the agent are conditionally independent of external states given the sensory states Markov blanket and, similarly, the external states are conditionally independent of the internal states given the active states of the Markov blanket. In simpler terms, the agent can only know about the world through its sensory states, and can only affect the world through its active states. This formalism captures the fundamental insularity of the organism without implying its isolation.

Markov blankets can be nested, such that cells have Markov blankets, organs have Markov blankets, and organisms have Markov blankets, and each level of organization can be seen as a higher-level active inference agent (Kirchhoff et al., 2018). The nested, self-organizing conception of biological agency has deep roots in the autopoietic tradition (Varela, 1991; Maturana and Varela, 1991; Varela et al., 1991), which first articulated the idea that living systems are defined by their capacity to produce and maintain their own boundaries — a notion that the Markov blanket formalism translates into precise mathematical terms. This structure, as we argue below, is particularly apt for modeling the mother–fetus/infant dyad, in which biological boundaries (notably the placenta) and informational boundaries are simultaneously at stake. The existence of a Markov blanket induces generalized synchrony between the agent and its environment over time, such that the agent’s internal states come to constitute a model of the external world — a formal expression of adaptive attunement (Friston, 2010).

### 2.4 The self-model and identity

Complex organisms require that their generative model includes a representation of the agent itself — a *self-model* — in order to distinguish between sensations produced by the agent’s own actions and those arising from independent external sources. This self-model is hierarchical, dynamic, and continuously updated; it encompasses current, past, and anticipated somatic and psychological states. In the AIF, identity can be understood as a coherent narrative structure that gives personal continuity and integrity to the agent’s inferential history, shaping the interpretation of new experiences and guiding action selection (Bouizegarene et al., 2020). The development of the self-model in early life — from the proto-self of the fetus to the increasingly articulated self-representation of the infant — is a central concern of the present theoretical framework.

## 3 The fetus/infant - mother as a co-evolving active inference dyadic system

### 3.1 The dyad as a coupled system of generative models

Attachment bonding, involving emotionally significant interactions between a child and caregiver, develops through sensory-motor exchanges with a variable flow of synchrony and mutual regulation. The organization of cognitive structures and attachment behaviors depends on factors like social context, caregiver responsiveness, and the child’s temperament.

**Figure 1:**
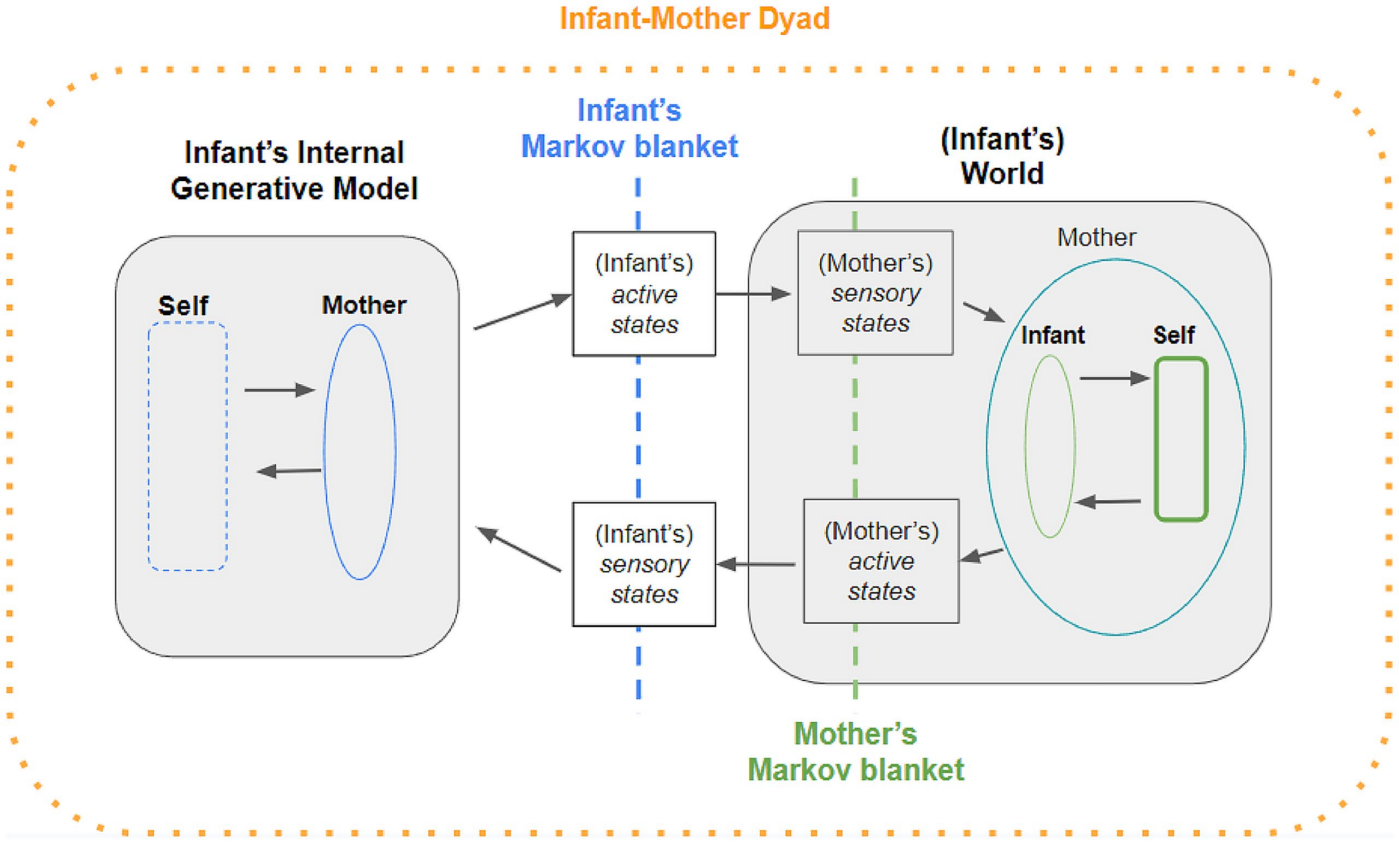
Generative model of a co-evolving dyad. In the figure, the mother represents the totality of the environment with which the infant interacts. The fetus is immersed in the mother’s body, which constitutes and filters every environmental stimulus. After birth, environmental elements other than the mother gradually become more significant. However, up until the period of defining an attachment style, the interaction with the mother and/or other primary attachment figures constitutes the main source of information for the formation of core models. These models, preverbal and predominantly procedural, will serve as a foundation for the processing and storage of future experiences regarding the characteristics of self and self in interaction with the world. In the picture, the asymmetrical nature of the dyad — whereby the child has only a proto-model of the self and the world, while the mother has a fully formed model of the world and a partially formed model of the child — is illustrated by the different thickness of the lines representing the self, the mother, and the infant (i.e., dashed line = proto, normal line = partially formed; thick line = fully formed).

We propose to model the mother–fetus/infant system as a dyad composed of two *coupled generative models*: two agents that co-evolve simultaneously but asymmetrically, each equipped with its own set of Markov blankets (Figure 1). This formulation builds on the nested nature of Markov blankets as a general feature of biological organization (Kirchhoff et al., 2018; Ciaunica et al., 2023). At the level of the dyad itself, the coupled system can be treated as a higher-order agent that pursues its own self-evidencing — minimizing free energy at the dyadic level to maintain the stability and coherence of the relationship. This dual-level organization (individual and dyadic) reflects the dynamic balance between preserving the individual viability of each member and sustaining the dyad as a functional whole.

The gestational period provides an especially striking instance of this co-evolutionary coupling. During gestation, proximity is not the goal of the fetus–mother relationship, as it becomes postnatally, but rather the condition of the fetus’s existence: the fetus is in a state of continuous co-embodiment (Ciaunica et al., 2021) with the mother, whose body constitutes and filters every sensory and metabolic input available to the developing organism. Previous analyses drawing on biology, physiology, and phenomenology have characterized pregnancy as an evolutionary form of relationship in which both mother and fetus exercise agency in the common interest of maintaining gestation (Quintero and De Jaegher, 2020; Lymer, 2011). In this view, the fetus–mother relationship is bidirectional and communicative: maternal movements and emotional states influence the fetus’s patterns of activity (Lymer, 2011) and, we suggest, the early calibration of its generative model. The continuous physiological transactions underlying gestation moreover give rise to a biological self-organization of the immune system that, along with neural organization, provides the substrate for a pre-cognitive, physiological distinction between self and non-self (Ciaunica et al., 2023).

Although attachment is caregiver-specific and children can form attachment relationships with multiple caregivers (Benoit, 2004; Boldt et al., 2014; Dagan and Sagi-Schwartz, 2021), the present work takes the mother as the primary caregiver reference in order to preserve continuity with the gestational period, during which the fetus is biologically and physiologically coupled specifically with the maternal organism. The dyadic composition of these two interconnected individuals is therefore adopted as the primary unit of theoretical analysis, while the role of additional caregivers is acknowledged as a limitation and avenue for future development.

### 3.2 Three defining characteristics of the dyadic system

*The caregiver as the dominant affordance environment*. The first defining characteristic of the model is that the caregiver occupies the majority — and during gestation, the totality — of the infant’s *affordance field*: the set of action possibilities that the environment offers to the agent (Gibson, 1977). This dominance can be understood as the condition in which the caregiver provides an organic niche for the development, physical nourishment, protection and also emotional and social support. Concretely, this characteristic implies that chemical signals, such as maternal hormonal secretions and those related to the composition of the foods she consumes, along with exteroceptive signals, like the mother’s voice and the beating of her heart, and the temperature of her body, constitute the sole sensorium during the prenatal period and the majority of the expected sensorium after birth and in the early months of life. The emotional exchanges related to postnatal interactions aimed at regulating and reassuring the child do not necessarily involve only the mother but also all other possible attachment figures. During gestation, however, the possibilities for action within the fetal sensorium are limited to the mother’s body and if the mother is subjected to consistently high levels of stress, the efficacy of the self-evidencing processes of the fetus in maintaining biological equilibrium, in terms of hormonal and physiological parameters, can be reduced. Thus, while the foundations of perceiving the environment as threatening or safe are laid during the gestational period – through the relationship of the fetus with the mother’s body – after birth, the organization of the child’s internal model depends on the outcomes of their agentive efforts, carried out through refined attachment behaviors. Notably, the child’s affordances differ markedly between the gestational phase and the prenatal phase: while in the period spent in the intrauterine environment, the possibilities for action primarily involve physiological adjustments and settling movements, after birth, the child assesses their agency based on the consequences of their attachment behaviors. In Figure 1 this is evident from the fact that, in the right side of the picture, the infant’s world is predominantly occupied by the mother. Therefore, the infant quickly builds and adaptively refines an internal model of the caregiver, and at the same time, a model of herself as an agent and of their relationship (see the left part of the figure where the child’s internal model is composed by the model of Self, the model of the mother, and their interactions). It is worth noting that, while the analysis here focuses on the mother as primary caregiver in line with classical attachment theory and the biological specificity of gestation, the theoretical framework applies equally to other attachment figures (fathers, adoptive parents, other consistent caregivers), whose role in structuring the infant’s affordance field and shaping its generative model deserves dedicated investigation (see Section 5, Limitations).

*The caregiver as a sentient agent with her own generative model*. The second salient characteristic of the model is that a large portion of the infant’s external “world” is itself a sentient agent – the mother- who is concurrently forming and adapting an internal model of the child. The caregiver forms and continuously updates representations of the infant, of herself as caregiver, and of their relationship (Koren-Karie et al., 2002; Meins et al., 2002). This is not merely a passive response to infant behavior but an active, predictive process: the caregiver anticipates the infant’s needs, assigns meanings to her signals, and organizes responses accordingly. In the right portion of Figure 1, this characteristic is represented by the mother’s models of herself, of the infant, and of their interactions. The modeling is a bidirectional process where both parties are engaged. Butthe models that the infant and caregiver hold of each other differ importantly in structure and depth (e.g., Stern, 1995; Alismail et al., 2022). The caregiver’s model of the child — rooted during pregnancy in somatic sensations of fetal movement and in future-oriented projections (Ammaniti, 1991; Smorti et al., 2020) — contributes to generate emotional states and physiological responses that in turn affect fetal intrauterine conditions, and -later- child’s internal state and behavior.

The mother’s representation of herself undergoes some changes during gestation and post-partum period, albeit generally to a lesser extent than her representation of the child, and this process likewise involves internal models of herself as caregiver, of the child, and of their emerging relationship. For example, a woman carrying an unwanted pregnancy will more likely have a negative representation of herself as a future mother, of the future child, of the anticipated relationship with the child, or of all three (e.g., Zimerman and Doan, 2003; Smorti et al., 2020). Such representations may contribute to chronic maternal stress, including psychological discomfort and associated biochemical variations (e.g., elevated cortisol levels), which in turn can affect the fetal intrauterine environment with significant physiological consequences (Zietlow et al., 2019). In this sense, the caregiver’s adaptive modeling of the child and of her Self-model contributes, from the outset, to the quality of the infant’s early inferential environment.

*The asymmetry of the co-evolving dyad*. The third characteristic of the model concerns the fact that the co-evolution of the internal generative models of the caregiver and the child is profoundly asymmetrical. The caregiver is an adult with a rich, extensively elaborated generative model of the world and a fully developed capacity for autonomous action. The infant – and even more so, the fetus – is at the earliest stages of generative model construction, with a proto-self that is only beginning to differentiate from the surrounding environment. In Figure 1, this is depicted by the child’s self-model being outlined by a dashed line, while the mother’s self-model is framed by a continuous line.

This asymmetry has two consequences of fundamental importance. First, the interactions with the caregiver have disproportionate power in shaping the architecture of the infant’s emerging generative model: the infant’s beliefs about the world — including beliefs about the reliability of others and about its own agentive efficacy — are formed primarily on the basis of what the caregiver does in response to the infant’s actions. Second, while the caregiver represents the privileged intentional target of the infant’s behavior and its primary source of validation, the infant occupies only a fraction of the caregiver’s full interactional world. The asymmetry progressively diminishes as the child develops and constructs an increasingly differentiated and comprehensive model of the world.

### 3.3 The Markov blanket structure of the dyad across development

The AIF framing of the co-developing dyad rests firmly on the notion of Markov blankets, which formally separates its internal states from external states while connecting them through sensory and active states. During gestation, we propose (following Ciaunica et al., 2023) that the placenta instantiates a physical structure with properties analogous to a Markov blanket: on the one hand separates the fetus from the maternal organism and, on the other hand, connects them, mediating the transfer of chemical information essential for the fetus’s self-organization and survival, and for the maintenance of pregnancy. The placenta, therefore, functions as both a physical and an informational demarcation between self and non-self, both for the mother and the fetus, allowing the fetus to self-organize coherently with the mother’s homeostatic and allostatic processes.

**Figure 2:**
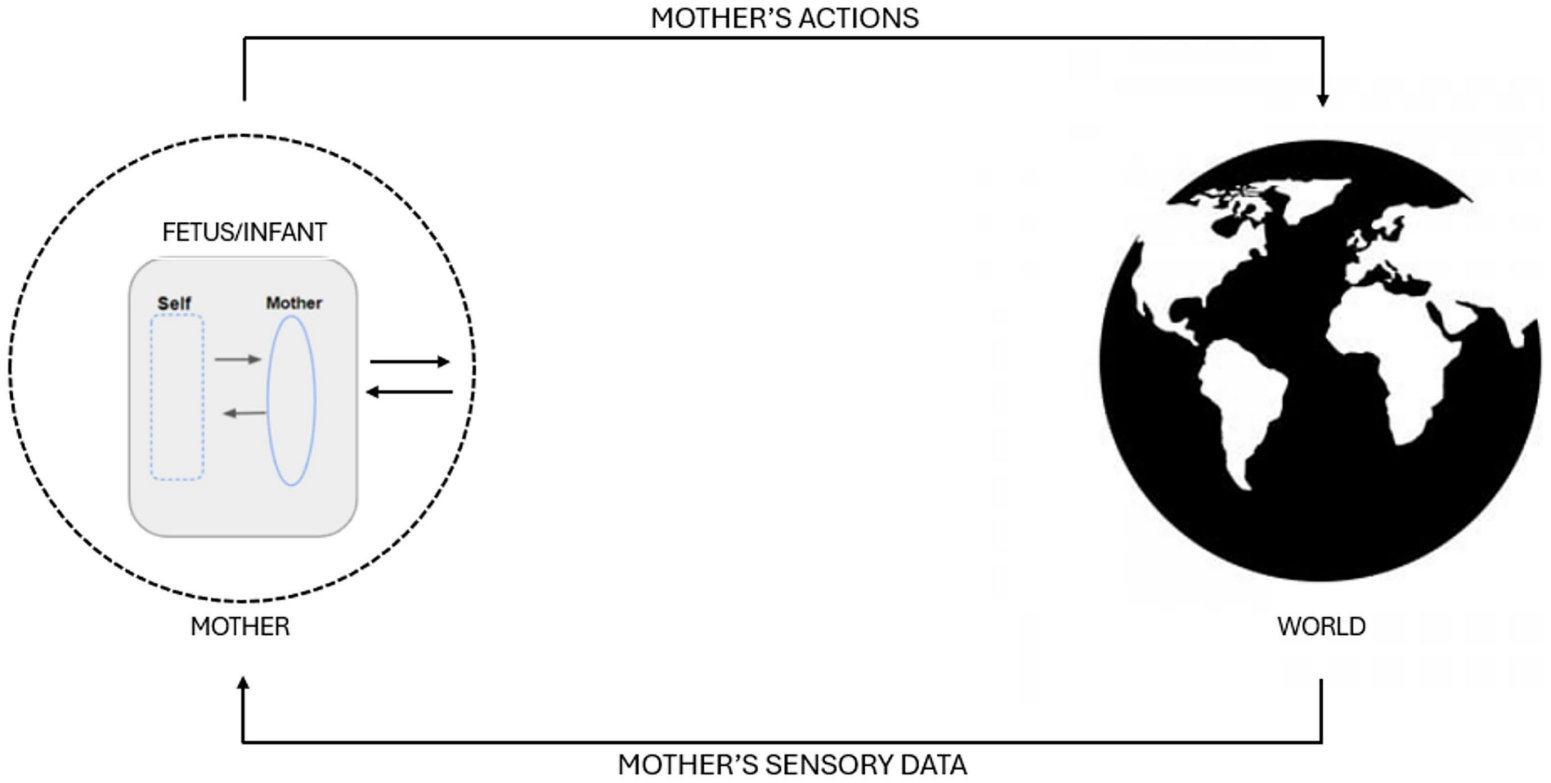
Interaction of the infant’s nested model with the external world exchanges between the fetus or infant and the external environment occur exclusively (in the case of the fetus) or mostly (in the case of the infant) conditioned on the mother’s actions and sensations.

After birth, the Markov blanket of the infant is reconstituted at a new boundary: the skin and the maternal breast now mediate the exchange of sensory information (tactile, olfactory, thermal information) and nutrients (breast milk, with its hormonal content), while the infant’s active states — principally crying, vocalizing, and motor behaviors — constitute the efferent channel through which the infant acts on the caregiver. The skin-to-skin contact and breastfeeding that characterize early postnatal life can be understood as a continuation, in a new form, of the intimate informational coupling that characterized gestation. In this sense, the mother can be described as performing Markov-blanket-like regulatory and filtering functions for the fetus/infant, both separating and connecting the fetus/infant system with the external environment. The emerging generative model of the fetus or infant may be viewed as developmentally scaffolded by, and partially nested within, the regulatory dynamics of the mother, providing a regulatory and interpretative infrastructure essential for the construction of the child’s expectations and Bayesian inferences (Figure 2).

A recent study (Bayne et al., 2023) suggests that neurophysiological evidence of brain activity indicating the integration of sensory inputs—consistent with perceptual processes—are present from birth and possibly even during the final stages of gestation. While the nature and extent of fetal perceptual experience remains genuinely uncertain and contested, these findings support the hypothesis that the fetus is not a passive recipient of maternal signals but an active, if rudimentary, participant in the inferential dynamics of the dyad. Converging evidence from recent empirical work further supports this view. Leisman et al. (2024) reviewed behavioral, neurobiological, and neuroimaging data indicating that between the second and third trimesters the fetal brain supports basic cognitive and behavioral processes — including sensory discrimination, habituation, and rudimentary anticipatory responses — that may scaffold the emergence of primary consciousness. Complementing this, Corcoran et al. (2023) proposed that fetal brain development is structured from the outset by bottom-up interoceptive inputs arising from spontaneous physiological rhythms such as the heartbeat, conceptualizing the emergence of cardiac self-regulation as an early instance of sensorimotor contingency learning that scaffolds the development of agentic control — precisely the kind of process that, in AIF terms, would constitute the earliest formation of a proto-self model. Reissland (2026) further reported that self-directed fetal movements, behavioral responses to external stimuli, and systematic changes in fetal facial expressions following specific sensory inputs collectively point toward the presence of elements of conscious states rather than purely reflexive activity. Taken together, these findings ground the concept of the fetal proto-self in an emerging empirical literature, while confirming that its nature and extent remain genuinely uncertain and require cautious interpretation. The increase in self-awareness across development can be linked to the progressive sharpening of the nested Markov blanket structure: during gestation, the boundaries between the fetal self-model and the maternal model are fuzzy and partially overlapping; after birth, they become increasingly well-differentiated as the infant’s generative model elaborates.

Figure 2 illustrates how the fetus/infant’s inferential model is nested within the mother’s, with maternal actions and sensory data mediating all exchanges between the infant and the broader world. This nested architecture is not merely a formal convenience: it reflects the biological reality that the infant’s environment is, in its most consequential aspects, the mother’s body and mind.

In conclusion, the present work sketches the main features of a dyadic generative model: the caregiver, who occupies the majority of the affordance field in which the child assesses their agency, is also an agent with its own model of self and the world, and the co-evolution of the dyad is asymmetric because the fetus/child is in the earliest stage of internal model formation while the mother has already an extensive experience in the world (although she may not have had the experience of carrying a child before). The synchronization between the activities of these two agents is a complex and dynamic process of precision estimation and precision weighting of their exchange signals (Friston and Frith, 2015) –The asymmetric relationship between mother and fetus/infant leads to a co-evolution of the two agents that results in the development of an attachment bonding and an attachment system in the child, in which the self-model component is strongly influenced by the behavior of the mother, particularly in response to the actions of the child. As proposed in this work, the mechanisms underlying these dynamics can be seen as underwritten by a principle of free-energy minimization, inferential in nature, and \ directed towards the self-organization of the agents that constitute the dyad, as well as the dyad itself. The introduction of this model is preparatory for a more specific discussion of some relevant aspects regarding the development of the attachment system, such as, firstly, the development of internal working models related to the characteristics of attachment experience.

## 4 Internal working models as generative models: a principled convergence

### 4.1 The structure of IWM and the generative model

Bowlby (1969/1982) described attachment behaviors as systems governed by a cognitive component, having two elements: instructions for achieving goals (proximity to the caregiver and a sense of security) and feedback mechanisms to adjust performance. Instructions are integrated into the control system as a consequence of its development within a specific environment and based on a certain genetic predisposition. In addition to instructions, the control system must have a representation or schematic map of the environmental topography to move safely, since it regulates behaviors whose expected outcomes require spatial localization over time. According to Young’s description of operational models, to which refers Bowlby (1969/1982), the brain creates facsimile models of the environment that can be manipulated and are used in the manipulation of the real environment. In addition to a model of the external world, it is necessary for the individual to also have a representation of their own agency in relation to it, and thus, a representation of the Self. The caregiver/world model and the self-model are distinct but influence and tend to confirm each other, contributing to predict the caregiver’s availability in times of need. Despite various alternative models of the same attachment figure or of the self that might be operative, there is always one dominant model, and generally, it is the relatively less conscious one (Bowlby, 1969/1982).

For a model to be a “good” model, it must be consistent with sensory observations, effective in new situations, extensible in imagination to cover potential realities that have never been experienced, and internally coherent (Bowlby, 1969/1982). As the accuracy and the comprehensiveness of the model increases, so do its predictive and adaptive capacities, respectively. Both the model of the world and the model of the Self are part of a complex biological control system and must be constantly updated, following Piaget (1936) principles of assimilation and accommodation. The structural parallels between IWM and the generative model of AIF are striking and, we argue, non-accidental. Both are hierarchically organized predictive structures; both include representations of the world and of the self; both are updated through experience according to principles of error minimization; and both are understood to develop across multiple timescales — from moment-to-moment physiological adjustments, through medium-term affective and cognitive reappraisal, to long-term structural changes in neural connectivity — with early attachment experiences shown to predict differences in amygdala reactivity, prefrontal regulatory capacity, and default mode network organization (Long et al., 2020) — and epigenetic organization — with the quality of early caregiving demonstrated to predict differential DNA methylation patterns at glucocorticoid receptor genes relevant to stress regulation and social behavior (Craig et al., 2021; Murgatroyd et al., 2015; Mulder et al., 2021). These multilevel findings converge on the view that IWM are not abstract cognitive structures but biologically instantiated models whose architecture is shaped, at multiple timescales, by the history of dyadic interaction.AIF, however, provides what IWM theory has historically lacked: a mathematically principled account of *how* models are updated, *why* certain environmental regularities are learned more readily than others, and *what* the formal consequences of model imprecision are for behavior and affect.

The embodied reformulation of IWM (Petters and Waters, 2014; Beckes et al., 2015) brings the two frameworks even closer. Rather than treating IWM as abstract symbolic representations, embodied reformulations shift the focus from viewing cognition as a purely abstract, substrate-independent symbolic computation (Shapiro and Spaulding, 2021) to response patterns grounded in bodily experience — patterns in which physiological, affective, and cognitive processes are mutually constitutive. Thus, IWM become embodied working models (Petters and Waters, 2014). In this perspective attachment experiences impact various temporal scales: in the short term, physiological variations occur, such as, e.g., changes in cortisol levels; in the medium term, the attribution of meaning to the experience takes place; finally, in the long term, there is a structural change in the IWM’s architecture that will influence information processing (Long et al., 2020; Petters and Waters, 2014). This view aligns naturally with the AIF perspective, in which the generative model is not a disembodied computational device but a biological structure whose predictions are grounded in interoceptive and exteroceptive sensory streams.

Atzil et al. (2018) add to the embodied reformulation of IWM, proposing that the learning of representations goes hand in hand with the allostatic organization of the organism, since dyadic interactions primarily serve allostatic regulation. Indeed, the caregiver’s proximity serves as an external regulator for the infant’s homeostatic and allostatic functions. The sensation of comfort or discomfort here is given by the association between the regulation of body parameters and related interoceptive sensations (Barrett et al., 2016). According to Fotopoulou and Tsakiris (2017), the significant other (i.e., the caregiver in attachment terms) acts as an external homeostatic and allostatic regulator for the infant, and their interaction allows the achievement of embodied mentalization, not only of one’s body but also of other’s bodies (such as that of the caregiver) (co-embodied mentalization). These proposals map onto the AIF account, in which the caregiver acts as a component of the infant’s effective environment that exogenously minimizes the infant’s free energy — a formal correlate of Bowlby’s “secure base.”

### 4.2 The prenatal roots of IWM: a hypothesis

In the second volume of his work, Bowlby (1973) suggests that the period during which attachment behavior is most easily activated and IWM are at their maximum plasticity goes from 6 months to 5 years of age. Before IWM becomes more rigid and automatic cognitive structures become established, there is a period of flexibility that extends into adolescence but gradually diminishes over time. Studies in cognitive and developmental psychology (for a review, see Sherman et al., 2015) have explored the fundamental cognitive abilities required for the development of IWM, asserting that the period of sensitivity for their formation broadly encompasses the first year of life, preceding the sixth month. Granier-Deferre et al. (2011) showed that fetuses exposed repeatedly to a melodic contour in the final weeks of gestation displayed a significant cardiac response to the same melody 1 month after birth, providing direct evidence of prenatal auditory memory and cross-temporal expectation. Kadic and Kurjak (2018) reviewed neurophysiological evidence indicating that the fetal brain supports forms of habituation, sensory discrimination, and rudimentary anticipatory responses from the third trimester onward. Delafield-Butt and Trevarthen (2013) further argued that the foundations of communicative intentionality are laid in prenatal motor development. Together, these findings suggest that IWM development may have prenatal roots.

We propose, as a theoretically grounded hypothesis, that the sensory and interoceptive experiences of the fetus within the maternal environment constitute the earliest form of generative model construction — a process of calibrating predictions about the characteristics of the self (proto-self) and the world (the mother’s body) that will scaffold subsequent postnatal IWM development. This proposal is consistent with the principle, articulated by himself Bowlby (1969/1982), that a working model must be capable of projecting into potential situations not yet experienced — precisely what would be required of a model that begins forming prenatally in anticipation of the postnatal environment. We acknowledge that this remains a hypothesis: direct empirical evidence for prenatal IWM formation in the sense intended by Bowlby is not yet available, and the cognitive capacities of the fetus are considerably more limited than those of the postnatal infant. The hypothesis generates, however, concrete testable predictions, some of which are discussed in Section 5.

## 5 The learned precision of the dyadic interaction as causal driver in determining attachment style

### 5.1 Precision learning and the organization of attachment

The attachment system is dynamic, aiming to maintain a steady state amid perturbations. Inconsistent caregiver responses lead to difficulties in predicting when and how needs will be met, causing hyperactivation of the attachment system and more rigid IWM (Cassidy and Berlin, 1994; Main et al., 2005; Pallini et al., 2019) and an insecure attachment system (Bowlby, 1969/1982; Main and Solomon, 1990). We propose that the key variable mediating between early caregiving experience and the development of a specific attachment style is the *learned precision of predictions* formed within the attachment relationship. Prediction precision, in the AIF sense, refers to the confidence the agent’s generative model assigns to its predictions — a quantity that is itself learned through repeated interactions and updated across developmental time. Consistent caregiving allows infants to build precise (i.e., reliable, confident) expectations about the consequences of its attachment behaviors — in particular, about whether proximity-seeking will succeed in securing caregiver availability. Inconsistent or unpredictable caregiving produces the opposite: a generative model whose predictions carry low precision, creating persistent uncertainty about the caregiver’s availability and the infant’s own agentive efficacy. For instance, a primary relationship in which caregivers convey conflicting affective messages and where availability alternates unpredictably with various degrees of unavailability will not allow a precise expectation about the outcome of the child’s queries, and consequently the formation of a confident attachment model.

We further propose that attachment style is organized along two dimensions that correspond to two distinct properties of the learned generative model:

The *axis of security* reflects the *content* of the learned predictions: whether the caregiver is predicted to be available and responsive (positive predictions → secure attachment) or unavailable and non-responsive (negative predictions → insecure attachment). This maps directly onto Ainsworth et al.’s (1978) original dimension of caregiver sensitivity.

The *axis of organization* pertains to the reliability of the predictions and depends on their *precision*. A precise model allows for reliable organization of attachment behaviors (high precision → organized attachment, either secure or avoidant), while an imprecise model leads to anxious/ambivalent organization or, at extremes, to disorganization of attachment. When predictions are imprecise, more cognitive resources are recruited in the effort to infer sensory inputs and behavioral consequences, complicating decision-making and facilitating higher levels of anxiety (Cassidy and Berlin, 1994; Main et al., 2005; Pallini et al., 2019).

### 5.2 Attachment style profiles as precision signatures

Within this framework, the three prototypical attachment styles can be interpreted as distinct configurations of model precision and content.

*Secure attachment* corresponds to a model characterized by high precision and positive content: the child confidently predicts that attachment behaviors will secure caregiver proximity and comfort. This confident prediction allows attention to be flexibly directed away from the attachment relationship toward the environment, enabling exploratory behavior (Main and Stadtman, 1981; Vandevivere et al., 2014).

*Anxious/ambivalent attachment* corresponds to a model with low precision and both positive and negative content: the caregiver’s responses have been sufficiently inconsistent that no highly reliable prediction can be formed. In this case, more cognitive resources are consumed in monitoring the relationship for evidence about caregiver availability, When a model is imprecise, it is difficult for the agent to settle incoming sensory signals at the expense of exploration. Into a meaningful interpretation, and to provide appropriate behavioral responses. The inability to resolve prediction errors generates chronic hyperactivation of the attachment system (Cassidy and Berlin, 1994) and a subjective experience of low self-efficacy — a persistent belief that one’s actions are insufficient to secure preferred states (Mikulincer, 1995).

*Avoidant attachment* presents a different signature: here, predictions about the caregiver have high precision, but negative content — a consistent history of unsuccessful proximity-seeking has generated a confident expectation of non-availability. In response, the child learns to suppress attachment behaviors and redirect attention toward the environment. The self-model in the avoidant child is, in this sense, functionally positive — agentive efficacy is attributed to autonomous, attachment-independent action rather than to dyadic interaction (Mikulincer, 1995). This learned model, while maladaptive outside the primary relationship and ineffective in resolving genuine threat, affords the model a good degree of precision in predicting its own agency.

These profiles have observable behavioral correlates. As attachment anxiety increases — that is, as model imprecision increases — behavioral policies shift toward increasingly desperate proximity-seeking, with markedly longer latencies to deactivate the attachment system after threat resolution (Ainsworth et al., 1978; Cassidy and Berlin, 1994). Conversely, avoidant children show attentional disengagement from attachment-relevant stimuli, consistent with the high-precision, negative-content model described above (Dan and Raz, 2012; Liu et al., 2017; Vandevivere et al., 2014).

### 5.3 Neurophysiological evidence for dyadic precision dynamics

Recent hyperscanning evidence lends neurophysiological support to the view that dyadic regulation depends on dynamic precision-weighting of interactional signals. A dual-EEG study by Capelli et al. (2026), using the Face-to-Face Still-Face paradigm, demonstrated that mother–infant neural synchrony changed significantly following interactional perturbation (the still-face), and was partially restored during the reunion phase. This pattern suggests that the dyad’s regulatory organization is not static but is continuously updated in response to disruptions in contingent exchange. Within the AIF perspective, such findings are interpretable as evidence that interactional disruption transiently increases the free energy of the dyadic system and reduces the precision of interactional predictions; repeated experiences of contingent repair subsequently contribute to restoring — and gradually strengthening — the precision of the infant’s expectations about caregiver responsiveness.

### 5.4 Prenatal origins of precision learning

We hypothesize that precision learning begins in the prenatal period, with the fetus calibrating its initial predictions on the basis of the mother’s self-regulatory patterns and stress responsivity. The regularity of certain maternal biological rhythms — including the sleep–wake cycle, hormonal patterns, and physiological responses to stress — constitutes the earliest statistical environment within which the fetal generative model is formed. Coherent and predictable maternal self-regulation would, on this hypothesis, promote the formation of precise initial priors; chronic stress or physiological dysregulation would introduce noise into the fetal sensorium, reducing the precision of early predictions (Sandman, 2015; Chang et al., 2010; Zietlow et al., 2019).

The genetic component provides a further dimension of this account. Several studies have identified genetic polymorphisms that predispose individuals to particular attachment styles or modulate their sensitivity to environmental influences (Bakermans-Kranenburg and van IJzendoorn, 2011; Luijk et al., 2011; Erkoreka et al., 2021). Within the AIF framework, we propose that such genetic variants may act by influencing the default precision-weighting of sensory signals — the prior degree of confidence assigned to incoming evidence before environmental statistics have been learned. So-called differential susceptibility genes (Bakermans-Kranenburg and van IJzendoorn, 2011) may amplify sensitivity to both negative and positive environments by raising the baseline precision attributed to prediction errors, thereby accelerating learning from experience in both directions. Risk and protective polymorphisms, conversely, may shift the default precision in ways that bias the trajectory of generative model development independently of specific environmental inputs. This remains a hypothesis requiring empirical investigation, but it provides a formally precise and testable account of gene–environment interaction in attachment development.

## 6 Attachment and exploratory behavior: pragmatic and epistemic policies

### 6.1 Attachment behaviors as pragmatic policies

Within the AIF, the behavioral repertoire of any agent can be classified according to the pathway of expected free energy minimization associated with each policy. *Pragmatic policies* are those that minimize expected free energy primarily by achieving preferred states — that is, by realizing biologically and psychologically valued outcomes. The behavioral sets constituting the attachment system — proximity-seeking, secure-base usage, reunion behaviors — are, on this account, pragmatic policies: their central goal is to achieve a state of proximity to the caregiver and thereby restore homeostatic and allostatic equilibrium. These policies correspond, metaphorically, to the function of the parasympathetic nervous system — restoring internal equilibrium after perturbation. This metaphor has physiological grounding in the well-documented bidirectional relationship between attachment experience and autonomic regulation: the quality of early attachment influences the parasympathetic nervous system’s capacity for physiological self-regulation, while the innate characteristics of the autonomic system influence the infant’s soothing susceptibility in social contexts (Oosterman et al., 2010; Tabachnick et al., 2021).

### 6.2 Exploratory behavior as epistemic policy

Bowlby (1969/1982) speaks of a system complementary to the attachment system, the exploration system, understood as a set of activities through which a child interacts with and learns from the environment. This behavior is closely linked to the presence of a primary caregiver who provides a “secure base,” since it is possible when attachment needs are satisfied and the child feels safe navigating the environment, knowing they can return to a safe haven at any time. In AIF terms, exploration is a set of *epistemic policies*: behavioral sequences whose primary function is the acquisition of novel information and the consequent reduction of uncertainty about the model of the world and the self. Epistemic policies — such as observation, problem-solving, and interaction with novelty — maximize the expected information gain of a course of action. They are activated under conditions of relative safety — when the level of uncertainty associated with the current state is low enough that the agent can afford to allocate resources to learning rather than to the urgent restoration of preferred states. The complementary relationship between attachment and exploration — Bowlby’s observation that a secure base enables confident exploration — maps precisely onto the AIF relationship between pragmatic and epistemic policies: when pragmatic security needs are satisfied, the expected free energy of epistemic policies falls below that of attachment policies, and exploration is preferentially selected. This provides a formal grounding for the intuition that attachment security enables curiosity and cognitive development.

### 6.3 Attachment style and the dysregulation of policy selection

Attachment style shapes the conditions under which these policy transitions occur, with predictable differences across the three prototypical styles.

In children with *secure attachment*, exploration is activated in conditions of objective safety, with ready deactivation of attachment behaviors when proximity to a sensitive caregiver is achieved. The flexible transition between pragmatic and epistemic policies reflects a well-calibrated, high-precision generative model.

In children with *anxious/ambivalent attachment*, the imprecision of the model means that safety signals are difficult to recognize unambiguously, and attachment policies remain active even in objectively safe conditions. Exploration is therefore suppressed not because danger is present but because the model cannot confidently classify the situation as safe. The result is a self-reinforcing pattern of proximity-seeking that limits epistemic exploration and constrains the development of a more differentiated model of the world (Cassidy and Berlin, 1994).

In children with *avoidant attachment*, a different dysregulation occurs. Because consistent caregiver non-availability has rendered the caregiver an unreliable agent of allostatic regulation, the child learns to suppress attachment behaviors even under threat — activating epistemic (exploratory) policies in conditions of danger as a strategy for autonomous internal state regulation. This is a formally interesting case in which epistemic policies are recruited as a substitute for failed pragmatic regulation, consistent with behavioral observations from the Strange Situation protocol (Ainsworth et al., 1978; Main and Stadtman, 1981).

### 6.4 Continuity with personality development

The differentiation of pragmatic and epistemic policy preferences established in the attachment period does not end there but constitutes, we propose, a developmental scaffold for later personality organization. Safron and DeYoung (2021) and Safron and Sheikhbahaee (2023) have described a cybernetic model of personality grounded in the Free Energy Principle, organized around two meta-traits: *Stability* (associated with the protection of pre-existing strategies from disruption and the maintenance of homeostatic and goal-directed coherence) and *Plasticity* (associated with exploration, updating, and adaptability in the face of novel challenges). We propose that the attachment-period learning of a preference for pragmatic security policies over epistemic exploration — or vice versa — constitutes a developmental precursor to these personality meta-traits, establishing a continuity between early relational experience and later dispositional orientations. This is a theoretical hypothesis that invites empirical investigation through longitudinal developmental research.

## 7 Discussion

### 7.1 Summary of the theoretical framework

This paper has proposed a theoretically grounded hypothesis: that the development of attachment styles emerges from the progressive construction, beginning in the gestational period, of a generative model in the fetus/infant that is shaped — in both its content and its precision — by the dyadic dynamics of the mother–fetus/infant relationship, operating under the organizing principle of free energy minimization.

The core theoretical contributions are as follows. First, we have reconceptualized the mother–fetus/infant dyad as a system of two asymmetrically co-evolving generative models coupled through a hierarchical Markov blanket structure that changes across developmental stages (gestation → birth → early infancy). Second, we have proposed that IWM are best understood as hierarchical generative models, and that the AIF provides the formal machinery needed to give Bowlby’s original control-theoretic intuitions computational and biological substance. Third, we have identified the learned precision of dyadic predictions as the proximal causal variable determining the organization of the attachment system, with attachment style emerging as a function of both the content and the precision of predictions formed in early caregiving relationships. Fourth, we have reinterpreted the attachment–exploration complementarity as a dynamic of pragmatic and epistemic policy selection modulated by the precision of the child’s current generative model.

### 7.2 Integration of multiple scientific perspectives

A primary motivation for this theoretical proposal is that it provides, starting from first principles, a common formal language for perspectives on attachment that have hitherto developed in relative isolation. Genetic research on attachment susceptibility (Bakermans-Kranenburg and van IJzendoorn, 2011; Luijk et al., 2011) can be reframed as evidence for genetically modulated precision-weighting. Epigenetic findings (Craig et al., 2021; Murgatroyd et al., 2015) can be understood as evidence that early precision dynamics — shaped by the caregiving environment — produce lasting changes in the biological substrate of the generative model. Neuroscientific evidence for attachment-related structural and functional neural changes (Perlini et al., 2019; Long et al., 2020; Schore, 2021; Choi et al., 2018) can be interpreted as the neural implementation of model updating at different timescales. Cognitive developmental findings on IWM organization (Sherman et al., 2015) are naturally accommodated by the hierarchical generative model construct. Recent hyperscanning evidence (Capelli et al., 2026) of interneural synchrony during responsive mother-infant interaction speaks directly to mutual attunement of the two generative models of the dyads across their Markov blankets.

This integrative capacity distinguishes the present proposal from other accounts that, while valuable, address only one or a few dimensions of the attachment phenomenon. At the same time, integration under a common framework does not constitute evidence for the framework: each of the mappings proposed above represents a theoretical interpretation that requires empirical evaluation, and alternative interpretations of the same evidence remain possible.

### 7.3 Empirical predictions and testable hypotheses

A strength of the AIF framework is that its formal precision generates concrete empirical predictions. We outline several that are particularly tractable:

1. *Prediction precision should be measurable as a dimension independent of security.* If the axes of security and organization correspond, respectively, to the content and the precision of predictions, then measures of prediction precision in the attachment domain (e.g., latency and consistency of caregiver-expectancy responses in infants) should predict disorganization independently of global security classification.
2. *Prenatal caregiving quality should predict neonatal prediction precision.* If precision learning begins prenatally, then measures of maternal self-regulation, stress reactivity, and behavioral predictability during pregnancy should correlate with indices of neonatal sensory expectancy and habituation — potential early markers of the initial generative model’s precision.
3. *Genetic susceptibility polymorphisms should moderate the relationship between caregiving precision and attachment organization.* Specifically, differential susceptibility variants should amplify both the positive effects of high-precision caregiving and the negative effects of low-precision caregiving on attachment organization.
4. *Computational modeling of dyadic interaction.* The AIF framework is sufficiently formalized to support agent-based computational simulations of mother–infant interaction, in which the precision of the caregiver’s policy signals can be systematically varied to generate predictions about emergent attachment patterns.

### 7.4 Clinical implications

While the primary contribution of this paper is theoretical, the framework has implications for clinical practice and intervention. The identification of precision learning as the key mechanism suggests that interventions targeting the consistency and predictability of caregiver responses — rather than their warmth or sensitivity alone — may be particularly effective for infants at risk of insecure or disorganized attachment. Programs that support maternal self-regulation during the prenatal period may, on this hypothesis, have downstream effects on the precision of the fetal generative model, with consequences for postnatal attachment development. These implications remain to be tested empirically, and caution is warranted in translating theoretical proposals directly into clinical recommendations.

### 7.5 Limitations

Several important limitations of the present framework should be acknowledged. First, the analysis has focused on the mother as primary caregiver, both because this is the focus of classical attachment theory and because the biological specificity of gestation (hormonal exposure, uterine environment, placental coupling) provides theoretically tractable grounding. However, attachment develops in relation to any consistent caregiver, including fathers, adoptive parents, and non-biological caregivers. Cultural variation in caregiving practices, caregiver roles, and the social embedding of the dyad represents a further dimension that the current framework does not address. Future theoretical development should extend the model to multi-caregiver systems and incorporate cross-cultural considerations.

Second, the claims regarding prenatal IWM formation and prenatal precision learning remain hypothetical. The cognitive and affective capacities of the fetus are genuinely uncertain, and the empirical evidence bearing on prenatal learning is suggestive rather than definitive. We have sought to mark these claims explicitly as hypotheses throughout the paper, but readers should be aware that direct empirical tests of the prenatal components of the model are not yet available.

Third, the intergenerational transmission of attachment style — whereby the attachment style of a parent predicts, with moderate accuracy, the attachment style of their child (Van Ijzendoorn and Bakermans-Kranenburg, 2019) — represents a major explanandum that the current framework is positioned to address (through the mother’s generative model and its influence on dyadic precision) but has not done so in detail. This is an important direction for future theoretical and empirical work.
